# Optical Fibre Pressure Sensors in Medical Applications

**DOI:** 10.3390/s150717115

**Published:** 2015-07-15

**Authors:** Sven Poeggel, Daniele Tosi, DineshBabu Duraibabu, Gabriel Leen, Deirdre McGrath, Elfed Lewis

**Affiliations:** 1 Optical Fibre Sensors Research Centre, University of Limerick, Limerick, Ireland; E-Mails: ddineshbabu@hotmail.com (D.D.); Gabriel.leen@ul.ie (G.L.); 2 School of Engineering, Nazarbayev University, Astana 010000, Kazakhstan; E-Mail: daniele.tosi@nu.edu.kz; 3 Graduate Entry Medical School, University of Limerick, Limerick, Ireland; E-Mail: Deirdre.McGrath@ul.ie

**Keywords:** optical fibre sensors; Fabry, Perot interferometer; pressure sensors

## Abstract

This article is focused on reviewing the current state-of-the-art of optical fibre pressure sensors for medical applications. Optical fibres have inherent advantages due to their small size, immunity to electromagnetic interferences and their suitability for remote monitoring and multiplexing. The small dimensions of optical fibre-based pressure sensors, together with being lightweight and flexible, mean that they are minimally invasive for many medical applications and, thus, particularly suited to *in vivo* measurement. This means that the sensor can be placed directly inside a patient, e.g., for urodynamic and cardiovascular assessment. This paper presents an overview of the recent developments in optical fibre-based pressure measurements with particular reference to these application areas.

## 1. Introduction

The pressure in a living human body is influenced by internal (e.g., muscles, fluids) and external (e.g., gravity, atmospheric) forces. The measurement of pressure and forces *in vivo* is a key asset in a range of biomedical applications, including cardiovascular and urologic diagnostic procedures, surgical procedures and monitoring of invasive treatments [1].

The most popular devices currently used for medical pressure measurement are based on catheters and guidewires. Air-charged catheters are low-cost and popular, particularly for urology applications [2]. Fluid-filled catheters represent a valuable alternative with a more stable response and are very popular in urology [3,4] and cardiovascular [5,6] applications. The work of Cooper *et al.* [3] compared the pressure response of air-charged and fluid-filled catheters. They demonstrated that air-charged catheters act as an overdamped system, whereas water-filled catheters act as an underdamped system. Pressure guidewires represent a modern alternative to catheters, having a smaller footprint and higher cost [7,8]. Guidewires and catheter pressure transducers are based on the principle of electro-mechanical pressure transducers [9–11]. Often, the pressure-sensing device is integrated in a complex catheterization, with multiple functionalities [12]. Commercial products are well established in urology and cardiovascular applications.

An alternative to these electro-mechanical pressure sensors, the optical fibre pressure sensor (OFPS) has become increasingly common in the medical field. This overview is intended for: (1) medical doctors to introduce them to the topic of OFPS; and (2) for engineers with a view toward understanding the demand for pressure sensor technology within the medical environment and the medical applications of current available technologies. The aim of this review therefore is to describe the use of the optical fibre pressure sensors applied in medicine with particular focus on the current state-of-the-art in technology and developments in the context of several applications, including those that are currently established and emerging in the medical field.

The paper is arranged as followed: Section 2 describes pressure measurement requirements within the relevant medical areas of interest (e.g., cardiology, urology, *etc.*) and the working principles of existing pressure sensors. Section 3 gives an introduction to optical fibre pressure sensors and provides details of the technology involved. Section 4 provides an overview of OFPS in biomedical applications and commercial products. Section 5 comprises a conclusion on the technologies reported.

## 2. Pressure Sensors in Medicine

**Figure 1:**
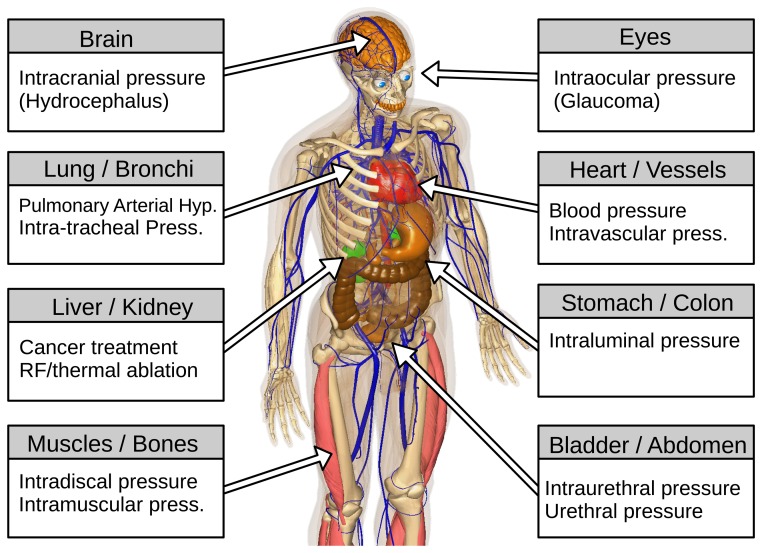
Body parts with pressure measurements and the relevant underlying physiological/pathophysiological condition associated with each organ/tissue (created in bodyparts3d [13,14]).

In the medical field, a sensor represents a device that responds to a physical stimulus and transmits a resulting impulse. Therefore, the fundamental purpose of a sensor system is to accurately measure a signal that enables the well being of a patient to be determined. The human and animal organism is a complex combination of a variety of organs, bones, joints and muscles (Figure 1 [13,14]). Each body part has its own set of characteristics (e.g., volume, structure, inner pressure, *etc.*). Additionally, each component may undergo a unique dynamic change in pressure, either due to normal physiological changes or as a result of an underlying pathophysiological process during the course of an illness. Clausen and Glott [15] recommended dividing the body pressures into three domains: (1) low pressure regions (e.g., capillaries and brain); (2) medium pressure regions (e.g., heart and lung); and (3) high pressure regions/states (e.g., joints and pressure changes during ablation techniques).

The requirements for a particular pressure sensor technology depend strongly on the area of interest (urology, cardiovascular, *etc.*), the place of measurement (e.g., organ, bone or muscle) or the method for which the sensor is employed (single point measurement, cancer treatment or long-term observation). Furthermore, any sensor or sensor system adopted for physiological measurement in the human body must meet certain fundamental standards of the suitability of use. Such standards are generally defined by an authorization institute, such as the Food and Drug Administration (FDA) and by the International Organization for Standardization (ISO), with particular reference to ISO 10993 [16] (Biological Evaluation of Medical Devices Part 1: Evaluation and Testing) and ISO 13485 [17] (Medical devices—Quality management systems—Requirements for regulatory purposes).

### 2.1. Technical Pressure Sensor Requirements

There are technical standards that must be met for each specific task, e.g., in the case of cardiology pressure analysis, standards defined by Association for the Advancement of Medical Instrumentation (AAMI) (in ISO 81060-2) [18], must be met when using sensors and devices in this setting. These standards are also FDA approved [19].

Range is the difference between the minimal to maximal pressure values measured in the body cavity. The pressure range can vary in normal physiological states from a large range 0–20 kPa (0–150 mmHg) in the case of left ventricular pressure to narrow ranges 0–1 kPa (0−7.5 mmHg) in the case of intra-cranial pressure. In diseased or pathophysiological states, pressure can fall as low as −10 kPa (−75 mmHg) in the case of intra-alveolar and intra-tracheal pressure and can rise as high as 40 kPa (300 mmHg) for aortic and left ventricular pressure. The diastolic pressure in a relaxed blood vessel has a normal range of 60–80 mmHg and can be elevated as high as 90–120 mmHg in a systolic contracted blood vessel [1].

The AAMI demands a pressure range of −4 kPa (−30 mmHg) to 40 kPa (300 mmHg) for a blood pressure transducer. Additionally, it should not be damaged with an overpressure in the range of −53 kPa (−400 mmHg) to 533 kPa (4000 mmHg) [20,21].

Accuracy (includes resolution), in terms of sensor requirements, often depends on the area of interest (e.g., heart, bone or muscle). A typical example arises in the case of blood pressure measurement, where for every 2.6-kPa (20 mmHg) increase in systolic pressure or 1.3 kPa (10 mmHg) in diastolic pressure, the mortality from ischemic heart disease and stroke doubles [22,23]. A small variation in blood pressure therefore can distinguish between a well, normotensive patient and a patient who is ill. For example, an adult can suffer from chronic disease (Table 1 [22]), where each stage is defined by a threshold of 133 Pa (1 mmHg). A high blood pressure value can be measured in proximal aorta. However, this blood pressure decreases as one moves away from the aorta to the femoral artery, radial artery and, subsequently, to the arterioles, becoming very small in the capillaries [24].

The American National Standards Institute (ANSI)/AAMI BP22:1994 (2006) therefore dictates that the accuracy for blood pressure measurement should be better than ±1% in the range of −4 kPa (–30 mmHg) to 6.7 kPa (50 mmHg) and ±3% in the range of 6.7 kPa (50 mmHg) to 40 kPa (300 mmHg) [21,23].

The sampling rate is the number of measurements acquired in one second and depends on the periodicity and waveform of the pressure signal. The fundamental natural frequency (f_n_) in a heartbeat rate up to $120\frac{\text{beats}}{\text{s}}$ is f_n_ = 0.5 Hz. However, the complex waveform requires further harmonics to rebuild the correct shape. With respect to Nyquist's theorem [25], a system should acquire at least double the highest frequency present in the signal, to preserve the signal. In practice, a medical sensor should acquire 5–10-times more samples than the highest frequency [26]. Further investigation allows an additional filtering and/or down sampling to analyse the important frequency band.

Since most internally-deployed sensors in medicine are placed in a catheter, with the function of either housing the sensor or working as a transducer, this may affect the frequency response. Gardner [27] demonstrated the affect of a catheter on the shape of a heart pulse in which the catheter is treated as a second-order system (with elasticity, mass and friction) having a natural frequency and damping factor. Gardner demonstrated that an overdamped catheter would not detect the dicrotic notch of a single heart beat, and an underdamped catheter would result in a noisily (incorrectly)-formed shape, thereby altering the accuracy of the pressure measurement. The AAMI therefore recommended a minimum frequency (200 Hz) for devices monitoring blood pressure [21].

Additional pressure sensor requirements are dependent on the specific task. For example, in small vessels, an *in vivo* pressure sensor with a large diameter can effect the measurement by restricting the blood flow. Furthermore, the magnetic field in a magnetic resonance imaging (MRI) can impair electrical sensors. In radio-frequency (RF) ablation techniques, the high intensity of electromagnetic radiation-generated temperature can impair and, in some cases, destroy the pressure sensor. Innovative adaptive techniques therefore are necessary in such circumstances. A list of requirements for specific medical fields are therefore shown in Table 2.

### 2.2. Principles of Pressure Sensors

The two principles of pressure measurement are exemplified by the classical strain gauge transducer and diaphragm displacement sensor [43], equally applicable in the case of optical fibre sensors (OFS). These principles will be explained in the following.

**Figure 2:**
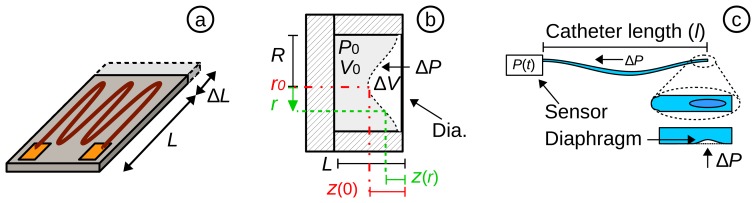
(**a**) Schematic of a piezoresistive sensor; (**b**) schematic of a diaphragm displacement sensor; (**c**) water-filled catheter as the pressure transducer.

Strain gauge transducers are characterised as those that exhibit a change in their output parameter in response to the measurand (*i.e.*, strain), e.g., electrical resistance (Figure 2a) or wavelength in optical sensors. The gauge factor (*i.e*., sensitivity), in Equation (1), is determined by the relative change of resistance (Δ*R*/*R*) with respect to the relative change of the length (Δ*L*/*L*) (also called strain *ϵ*). In an electrical sensor, the change in resistance can be most effectively measured using a Wheatstone bridge [44,45].

(1) $$\text{GF}=\frac{\Delta R/R}{\Delta L/L}=\frac{\Delta R/R}{\epsilon }$$

Diaphragm displacement sensors are based on micro-electromechanical systems (MEMS) technology, in which sensors have a bendable flat surface (*i.e.*, the diaphragm) on a sealed cavity. The diaphragm bends (deforms) according to the change of pressure and can be capacitance based or based on a piezoelectric transducer. The sensor structure is shown schematically in Figure 2b. In the initial state, the cavity has an initial volume (*V_0_*) and pressure (*P_0_*). Since the cavity is sealed, a change in pressure (Δ*P*) causes the medium (e.g., air) inside the cavity to compress/expand (Δ*V*).

In the case of a circular cross-section diaphragm with clamped edges, the bending of the diaphragm (*z*(*r*)) can be theoretically predicted using Equation (2), provided the bending displacement is limited to less than 30% of the diaphragm thickness (*h*). This principle is used for capacitive and piezoresistive sensors. In the centre of the diaphragm (*r* = 0 = *r_0_*), the bending displacement is at maximum. Whereas on the clamped edge (*r* = *R*), no bending occurs (*z*(*R*) = 0). The elasticity depends on Poisson's ratio (*μ*) and Young's modulus (*E*) of the diaphragm material. The displacement of the diaphragm can be measured by a frequency-excited gain circuit [1].

(2) $$z\left(r\right)=\frac{3}{16}\cdot \frac{\left(1-\mu^{2}\right)\left(R^{2}-r^{2}\right)}{Eh^{3}}\cdot \Delta P$$

Catheter-based pressure systems use a catheter filled with an incompressible medium (e.g., water or saline solution), connected to a pressure sensor (Figure 2c). A change in pressure at the tip of the catheter results in a deformation of the diaphragm, which compresses or decompresses the water and, hence, transmits the pressure directly to the connected sensor. The natural frequency (*f_0_*) of a water-filled catheter (with a density of *ρ*) can be calculated by Equation (3). Togawa *et al.* [1] demonstrated the limitation of the water-filled transducer. For standard-sized medical catheters [46,47], with an outer diameter (o.d.) *r_0_* = 1.66 mm (5 Fr), an inner diameter (i.d.) of *r_i_* = 0.66 mm (2 Fr) and a length of *l* = 1.25 m connected to a standard pressure sensor with an elastance of $K=3.3\cdot 10^{14}\frac{\text{Pa}}{{\text{m}}^{3}}$, the theoretical natural frequency is estimated at (*f_0_* = 48 Hz) [1].

(3) $$f_{0}=\frac{1}{2\pi }\sqrt{\frac{k}{m}}=\frac{r_{i}}{2}\sqrt{\frac{K}{\pi \rho l}}$$

Volume-restricted areas (e.g., brain or miniature blood vessels) demand thin catheters. Ultra-thin catheters (*i.e.*, microtubing, Johnson Matthey) have an o.d. of 0.3 mm and an i.d. of 0.254 mm (*i.e.*, P.N.24468A) [48]. Replacing the previously mentioned catheter with the ultra-thin catheter ( $r_{i2}=\frac{1}{3}r_{i}$) and assuming the same length, the natural frequency would decrease to $f_{02}=\frac{1}{9}f_{0}$ (*i.e.*, *f_02_* = 5.3 Hz). Considering the presence of additional compressible air-bubbles trapped in the catheter structure, damping and reducing the natural frequency [49], it is necessary to locate the sensor at the tip of the catheter or at least very close to it. In the case of very small catheters (below 1 Fr), catheter-tip sensors, such as optical fibres, can uniquely fulfil these requirements.

### 2.3. Commercial Sensors

The specifications of some commercially available electrical sensors [50–53], which are currently used in medicine, are included in Table 3. Recently, the FDA approved the first implantable wireless sensor for pulmonary artery pressure measurements [54], by CardioMEMS™.

## 3. Optical Fibre Pressure Sensors

From the 1960s to the early 21st century, optical fibre sensors were largely based on intensity modulation, and this was the dominant technology for use in OFS, at the time being based on a simple architecture and low-cost interrogation. They therefore represented a potential alternative to the standard medical pressure sensors [55].

### 3.1. The Development of Optical Fibre Sensors

Many early-stage OFSs were based on optical fibre bundles [56,57], which were implemented in a single fibre oriented to a distal reflective mirror [58,59], with a two (or more)-fibre system [60] or a fibre modulated by an internal cavity [61]. Several patents regarding intensity-modulated OFSs were filed from the 1990s onward [62–65], complementing existing patents for catheters to host such sensors, such as Purdy *et al.* [66]. The Camino sensor, industrialized by Integra LifeSciences, represents an important commercially available product and is currently a standard system for the measurement of intra-cranial pressure (ICP) [67–69]. OFS based on a microbending principle (*i.e.*, attenuation of intensity) [70,71] were recently adopted in the pressure measurement area. Intensity-modulated OFS are relatively simple in design and, hence, relatively low cost, but suffer from long-term instability. Changes, such as the variation of the received optical intensity due to source output power drifts, fibre movements, or the degradation (*i.e.*, ageing) of components in the system, or on the fibre tip contribute to error in the measured pressure signal.

Following the advent of the fibre Bragg grating (FBG) sensors (in the 1990s), much research effort was dedicated to applying the emerging FBG technologies to pressure sensing in the medical environment. FBGs are highly sensitive to strain, and due to recent developments in draw-tower fabrication techniques [72,73] can be manufactured as bend-insensitive fibre structures with excellent tensile strength, on standard 125-μm diameter, as well as 80-μm fibres. A major technical challenge of implementing FBGs as pressure sensors is the physical conversion from pressure to strain, which is the fundamental parameter measured by the FBG. FBG sensing systems are capable of providing a solution to the problem of intensity-modulated OFS, as they provide intensity-independent output.

Within the last seven years, due mainly to improvements in microfabrication technologies (e.g., the availability of splicers, lasers and hybrid electro/optic facilities for optical fibres), cavity structures based on Fabry–Perot principles have become realisable and represent a major research focus in pressure sensing technology. In this context, the Extrinsic Fabry–Perot interferometer (EFPI) represents a significant technological advancement and is based on a sound principle of operation, which is a wavelength-dependent intensity variation resulting in a spectral shift [74]. EFPI sensors have overcome the main drawbacks of intensity-modulated OFS and FBG sensors, in so far as they provide intensity-independent detection, but sensitivity and accuracy are more than three orders of magnitude greater than FBGs.

### 3.2. Advantages of Optical Fibre Sensors

Sensors based on optical fibres are emerging as an excellent alternative to electrical sensors based on catheters, guidewires and MEMS [75]. For medical applications, OFS have many strategic advantages over classical measurement techniques:

**Footprint and geometry:** Silica glass-based optical fibres used in most sensors are very small, since they have a 125-μm diameter (e.g., SMF-28 fibres), and optical fibres of an 80-μm diameter are currently commercially available. Furthermore, recently developed draw-tower fabrication of fibres and sensors [76] now allows the fabrication of in-fibre sensors without removing the fibre coating and, therefore, maintaining the tensile strength of the buffered fibre, which is critical when being placed in a range of environments. In addition, the broad availability of bend-insensitive fibres allows the operation of these sensors, even in the presence of tight bending. Commercial catheters have typically 4–6 Fr diameters ( $1\text{Fr}=\frac{1}{3}\text{mm}$), where a plurality of optical fibre pressure sensors can fit inside an individual catheter or guidewire.
**Distribution and integration:** OFS enable the detection of physical parameters (strain, temperature) at several points along a single fibre. This arrangement is extremely common with FBG sensors, using a wavelength division multiplexing (WDM) approach [76–80]. Recently, distributed sensing systems based on Rayleigh backscattering demodulation [81,82] have achieved a spatial resolution better than 1 mm. In addition, with a WDM approach, it is possible to integrate on the same fibre, at the sensing point, a plurality of sensors: a popular arrangement involves the integration of pressure and temperature sensor on the same fibre tip [83–86].
**Long-term capabilities:** As one of the main trends in biomedical science is to perform long-term diagnostics with minimally-invasive devices, the stability of pressure recording in the long term needs to be guaranteed. OFS guarantee an excellent stability rate, achieving typical stability of $1\frac{\text{mmHg}}{\text{hour}}$ with sensor prototypes [87] and about 3 mmHg/28 days in commercial sensors [88].
**Thermal properties:** The accuracy of pressure sensing technologies is also limited by cross-sensitivity to temperature variations. In the case of many sensors (e.g., electronic, mechanical), a temperature change has a non-linear effect on the output, which can only be mitigated by thermally-insensitive packages. In OFS, however, temperature and pressure dependences are both linear and can be mutually compensated [83].
**Total immunity to external EMI:** OFS can be fabricated from silica glass, which is a dielectric material and is inherently immune to electromagnetic interference (EMI). This makes OFS-based device compatible with the medical environment where EMI is central to a range of diagnostic and treatment techniques, such as as MRI, computed tomography (CT) scan, RF/microwave thermal ablation and other imaging and invasive medical procedures [70,89].

### 3.3. Theory and Working Principles

For the medical field, OFS can be grouped according to: (1) the placement area of the sensor (e.g., *in vivo, ex vivo, in vitro*); (2) the amount of usage (*i.e.*, disposal or reusable); or (3) the pressure range being recorded (low, middle, high). From a technical point, the OFS may be grouped according to structure, modulation and measurement.

**Figure 3:**
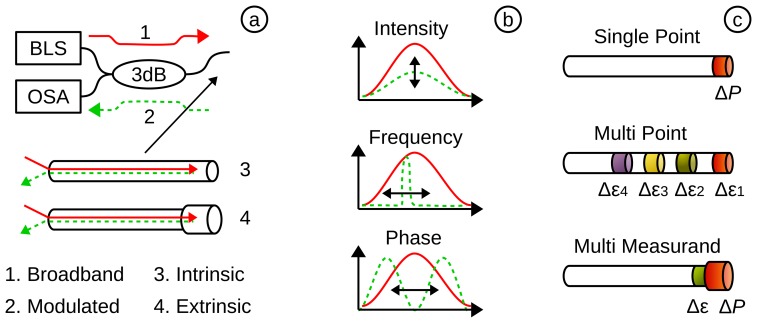
(**a**) The full sensor system based on a broadband light source (BLS), an optical spectrum analyser (OSA) and an intrinsic or extrinsic sensor; (**b**) the signal can be modulated by a change in intensity, frequency or phase; (**c**) the sensor may measure in a single or multi-point and acquire single or multiple measurands.

The structure of an OFS (Figure 3a) can be divided into intrinsic (*i.e.*, the sensing element is inside of the fibre) or extrinsic (*i.e.*, the fibre is extended with an external sensing element). It also shows the set-up with broadband light source (BLS), which emits a continuous spectrum (1) to the sensing element. There, the signal is modulated (2) by the measurand and travels back though a 3-dB coupler to the optical spectrum analyser (OSA). The advantages of reflection mode (as shown in Figure 3a) as opposed to transmission are that the sensor may be used as a reflective probe and the tip of the sensor can be inserted into the patient.

**The modulation** for OFPS (Figure 3b) may change: (i) the received intensity (*i.e.*, amplitude of the electric field E) by a displacement of a reflecting surface (e.g., diaphragm) or a bending of a fibre (*i.e.*, attenuation); (ii) the frequency (*i.e.*, wavelength λ) by a change of a the period of the grating (FBG); or (iii) based on a phase modulation (*ϕ*) (*i.e.*, interferometry by Mach Zehnder, Michelson [90,91] or Fabry-Perot [92]) using the phase property of the light.

**The measurement** for an OFS (Figure 3c) can depend on: (i) the point of interest (*i.e.*, single point, multi-point or distributed sensing); or (ii) the measurand itself (e.g., pressure (*P*), temperature (*T*) or both). It is also possible to extend the OFS to measure other measurands (e.g., from strain (*ϵ*) to pressure (*P*)).

### 3.4. Fabry-Perot Interferometer and Fibre Bragg Grating

The remit of this paper is a review of OFPS based on EFPI and FBG techniques. A more comprehensive review of optical fibre intensity-modulated sensors is available in Roriz *et al.* [55]. A recent review of medical FBG sensors is available in Mishra *et al.* [93] and for Fabry-Perot interferometer (FPI) sensors from Roriz *et al.* [94]. Both sensor types are based on the same principles shown in Equation (1) for the strain gauge and Equation (2) for the diaphragm displacement and can be used both directly and indirectly to measure the pressure. The recently investigated OFPS highlighted in this review are based on both principles and represent a major step forward in the current state of the art.

**Figure 4:**
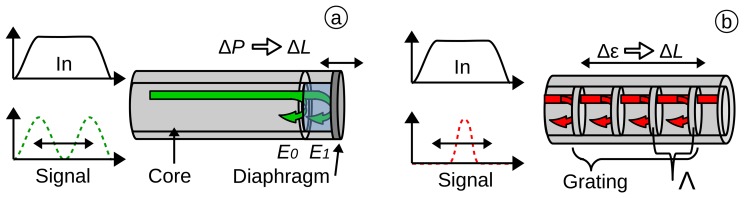
Reflected light in: (**a**) a low finesse Fabry–Perot interferometer (FPI) sensor and (**b**) in a FBG sensor.

**The Fabry-Perot interferometer** is based on the principle of interferometry [92,95]. The Fabry-Perot (FP) cavity is usually located on the tip of an optical fibre and enclosed by a miniature glass diaphragm [74]. The light from the source is coherent in the vicinity of the sensor and can potentially interfere with itself, e.g., by reflection. For a low finesse interferometer (Figure 4a), approximately 4% of the light intensity is reflected at the end face of the single-mode fibre (SMF). The residual light travels the length (*L*) to the diaphragm, where upon it is also reflected (e.g., another 4% for a glass diaphragm), travels back the same way and finally penetrates back into the SMF. Since the light from the diaphragm has experienced an additional path length (2*L*) through the cavity filled with air (refractive index (*n_0_*)), it has a phase difference, given by Φ_0_. The intensity (*I*) in the spectrum for each wavelength (λ) therefore depends on the distance of the diaphragm (*L*) to the SMF, shown in Equation (4) [96]. The change of the diaphragm with reference to the pressure (*i.e.*, Δ*L*(Δ*P*)), can be calculated in the same way as previously demonstrated in Equation (2).

(4) $$I\left(\lambda \right)=I_{1}+I_{2}+2\sqrt{I_{1}I_{2}}\cdot \cos\left(\frac{4\pi \cdot L\cdot n_{0}}{\lambda }+\Phi_{0}\right)$$

Alternative solutions for a diaphragm make use of bendable organic material to increase the sensitivity [97], ultra-thin metal diaphragms to improve the reflectivity [98] and a graphene membrane [99], as well as a polymer diaphragm sealed with a ultra violet (UV) mould [84]. Bremer *et al.* [83] demonstrated an EFPI sensors with an integrated FBG sensor for dual pressure/temperature detection and mutual compensation. Bae *et al.* [100] in fact proposed a multi-cavity approach for dual sensing. Their high sensitivity and accuracy, as well as their dual sensing versatility have resulted in commercial systems based on EFPI technology gaining momentum and an increasing market share [88,101,102].

**The fibre Bragg grating** is formed as a periodic change of the refractive index n_co_ (the core refractive index) with a distance of Λ (the pitch of the grating). The light is partially reflected at each grating period. This results in a narrow-band reflection at the Bragg wavelength (λ _B_), as shown in Equation (5), where n_eff_ is the effective refractive index of the optical fibres core in the region of the grating. With applied strain (*ϵ*), the pitch of the grating changes and, therefore, the Bragg-wavelength.

(5) $$\lambda_{\text{B}}=2{\text{n}}_{\text{eff}}\cdot \Lambda$$

The challenge is to measure the pressure and convert it into mechanical strain, so that it may be measured by the FBG. Kanellos *et al.* proposed a pressure sensor based on four FBGs based on a flexible patch [103]. Ahmad *et al.* [104] proposed a similar form factor. Another working principle proposed by Zhang *et al.* [105] was based on a piston-like architecture. FBG-based sensors are also gathering significant traction in novel robotic micro-surgery systems [106,107] for the measurement of axial and lateral contact force.

## 4. Optical Fibre Pressure Sensors in Medicine

Optical fibre sensors have been proposed for biomedical applications (e.g., *in vivo*, *ex vivo* or *in vitro*) for several decades. Early investigations of *in vivo* applications were undertaken [108], and many reviews have recently been compiled to evaluate their advantages in the medical field [109–115]. It is therefore the role of this review to highlight the use of optical fibre pressure sensors in the state-of-the-art medical practice.

### 4.1. Introduction

Lindström [59] proposed the first medical optical fibre pressure sensor in 1970. In 1984, Peterson and Vurek [116] analysed the utilisation of optical fibres. Nowadays, optical fibres can be fabricated from a wide range of different materials (e.g., plastic, chalcogenide glass), whereas silica glass-based fibres are known to have good bio-compatibility. Hench and Wilson [117] initially reviewed the compatibility of silica in 1986. This study was extended for silica-based MEMS [118,119] and finally tested for long-term in vivo implantation [120,121]. Yang *et al.* [122] (2003) tested OFS-based pressure sensors for 12 weeks and demonstrated their bio-compatibility and usability for *in vivo* human applications.

**Figure 5:**
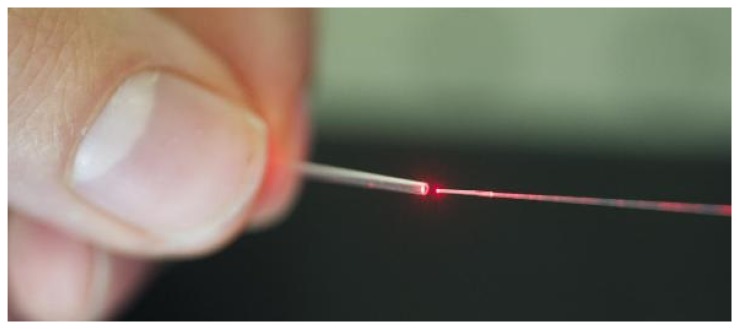
OFPS (**right**) illuminated in red and placed into a miniaturized catheter (**left**).

As previously outlined, modern optical fibre sensing technologies provide many advantages over non-OFS technologies when used for medical application. Their small size (Figure 5) makes them ideal for use in volume-restricted areas. Since they are fabricated from silica glass, they are immune to RF and, therefore, compatible for use in MRI. OFS (a Furukawa company) offers bio-compatible fibres for medical application. These fibres are bend resistant and also offer bio-compatible coatings, certified by North American Science Associates, Inc. (NAMSA) with ISO 10993 [123]. Optical sensors can also be sterilized, without affecting their properties. Stolov *et al.* [124] demonstrated the possibility of: (i) steam sterilization; (ii) ethylene oxide and (iii) gamma radiation on optical fibres with: (1) dual acrylate; (2) polyimide; (3) silicone polyether ether ketone (PEEK); and (4) fluoroacrylate hard cladding ethylene tetrafluoroethylene (ETFE).

### 4.2. Research on Medical Optical Pressure Sensors: In Vivo

In this review, OFPS are presented, which are used in the medical field for both *in vivo* and *ex vivo* applications as outlined in the literature. In some cases, the available literature is minimal; in other cases, there is extensive and detailed information available on the relevant sensors. Some typical medical application areas are highlighted below in which the use of OFS has proven successful.

**Gastroenterology:** 60–70 million Americans are affected by Gastrointestinal conditions every year [125]. One of the most common gastrointestinal symptoms is abdominal pain, which results in hospitalization in 15.9 million cases [126]. The magnitude of this medical condition and the implications for the health economy dictate the need for an inexpensive method of investigating the gastrointestinal tract.

In 2007, Takeuchi *et al.* [127] published a study on pharyngeal manometry. They used an FPI-based pharyngeal manometric sensor for deglutition analysis. The sensor (FOP-MIV, FISO Technologies) showed good agreement with their catheter-type reference sensor (P37-4109C05, Zinetics). The whole catheter structure was of a small size of only 2.08 mm in diameter, covering a pressure range of 30 kPa–30 kPa and a sampling frequency of 250 Hz. The sensor demonstrated a correlation factor of 0.999 to the reference sensor, and furthermore, a trace of a period of *in vivo* swallowing processes was recorded online and demonstrated good agreement with the reference instruments. The same sensor type (FOP-F125, FISO) was used by Kong *et al.* [128] (2013) for oesophageal variceal pressure measurements in three different patients.

**Figure 6:**
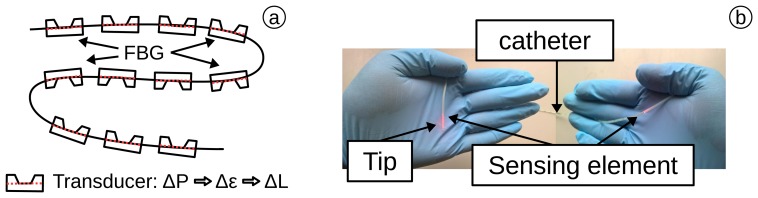
(**a**) Distributed pressure sensor based on an FBG chain with transducers; (**b**) sensor placed in a catheter.

Arkwright *et al.* [129,130] (2009) presented a manometry catheter based on FBG arrays (from FBGs) with a series of 72 sensing elements with a spacial distance of 1 cm. Each FBG is inserted into a casing design (schematic in Figure 6a) and is used to transpose pressure into strain, resulting in multi-pressure measurements [131,132]. The sensor structure was tested for 0–26.7 kPa (0–200 mmHg) with an accuracy of 0.4 kPa (3.1 mmHg), a sensitivity of $-0.001\frac{\text{nm}}{\text{mmHg}}$ and a frequency of 10 Hz. The FBGs are surrounded by a transducer, converting the pressure into strain (Figure 6a). The catheter (Figure 6b) was tested *in vivo* in a human colon over 24 h. In their study, they placed the sensing elements in the ascending colon, in the transverse colon, in the descending colon and in the sigmoid colon [129]. This successful test revealed the complex pressure nature of the colon for the first time. This technique is an example of where optical fibres have surpassed the gold-standard and, in fact, opened a new area of high-resolution manometry (HRM). This technique was recently (2014) used in a study of 10 healthy humans and revealed a new understanding in the propagating motor pattern of the human colon [133].

**Cardiology:** The recent report (2014/2015) of the American Heart Association (AHA) [134,135] revealed that in 2011, *ca.* 600,000 Americans died as a result of heart disease with a cost (direct and indirect) of 215.6 billion $. This again demonstrates the need to introduce cost saving methods within the healthcare system without compromising the quality of patient care.

The first optical sensor for intra-vascular pressure measurement was developed and clinically tested by Lindström *et al.* [59] in 1970. This intensity-based sensor was instrumental in optical sensors achieving successful entry into medicine. In 2002, Reesink *et al.* [136] published a feasibility study, using fibre-optic systems (Model 40EC, RJC enterprise) for invasive blood pressure measurements. They compared the OFPS *in vitro* to the gold-standard Millar (SPC-320 with a bridge amplifier), Baxter (uniflow external pressure transducer) and Sentron devices. Further tests *in vivo* in two goats followed. They demonstrated the high similarity of the OFPS to these gold-standard sensors. In 2003, Woldbaek *et al.* [137] described the use of an OFPS for pressure recording in the cardiology setting. They used an optical sensor (Samba) for pressure measurements in mice. They tested the sensors (o.d. 0.42 mm) in vitro with a drift of $<60\frac{\text{Pa}}{\text{h}}(<0.45\frac{\text{mmHg}}{\text{h}})$ and a temperature sensitivity of only $∼9\frac{\text{Pa}}{\text{k}}(∼0.07\frac{\text{mmHg}}{\text{k}})$ in the range of 22–37 °C. With a frequency response of 0–200 Hz, this sensor fulfils the technical specification. They inserted the OFPS in 18 male mice, into the left carotid artery to measure the aortic pressure (AP) and heart rate continuously. The optical sensor demonstrated good functionality, and Woldbaek *et al.* [137] concluded that this sensor is suitable for blood pressure measurement, even in small vessels.

Schreuder *et al.* [138] (2005) published a new measurement method using automatic intra-aortic balloon pumping (IABP) with a dicrotic notch prediction algorithm. For their evaluation, they applied an optical sensor (they refer to the same one that Reesink *et al.* used) in 27 patients with low ejection fraction (*i.e.*, undergoing cardiac surgery) for 20–48 h. The optical fibre sensor, combined with their novel algorithm, allowed a fully-automatic IABP timing. In the same year (2005), Pinet *et al.* [139] proposed that their OFPS (FISO) based on a micro-optical mechanical systems (MOMS) could also be used for IABP. A detailed analysis of the effect of fibre-optic IABP therapy on clinical management was performed by Yarham *et al.* [140]. Their FOP-MIV is now also FDA approved. Furthermore, a patent for an intra-aortic balloon catheter with a dual pressure sensor technology was filed in 2007 [141]. Mulholland et al. [142] (2012) reported the insertion of an 8-Fr, 50-cc SensationPlus™ intra-aortic balloon (IAB) catheter (Maquet Cardiocascular) in a 53-year-old man. As a result, the catheter expands the patients vessel, which results in greater diastolic blood volume, and the sensor supported more accurate monitoring.

Wu *et al.* [143] (2013) developed an optical sensor and used it in conjunction with a fractional flow reserve (FFR) technique in a swine model. In the last two years (2014–2015), the amount of research in this field has rapidly increased [144–148]. In 2014, Rodriguez *et al.* [149,150] used an OFPS (OPP-M, Opsens) for simultaneous pressure and volume measurement. The immunity to electromagnetic fields made it possible to use the sensor in an MRI during a left ventricle function assessment while undertaking an *in vivo* experiment in a ewe. The results were compared to the Millar sensor and demonstrated good correlation.

**Neurology:** A significant increase in ICP can be life threatening for neurological patients [151]. This can be caused by disease (e.g., cancer growth or accumulation of blood) or factors, such as blasts from explosions (e.g., improvised explosive devices (IEDs)). To confirm raised intra-cranial pressure, optical fibres can play a decisive role.

**Figure 7:**
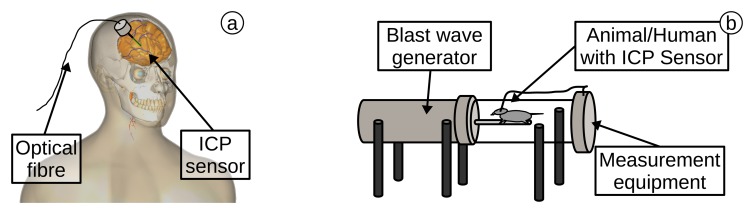
(**a**) ICP sensor, inserted into brain (created in bodyparts3d [13,14]); (**b**) schematic of blast wave generator with animal and ICP sensor inside.

In 1996, Shapiro *et al.* [152] demonstrated intra-parenchymal cerebral pressure monitoring in 244 patients (e.g., with intra-cerebral pathology, including trauma and intra-cerebral haemorrhage), using OFS technology. The measurements were performed from 1988–1993 with an average time of seven days (up to 24 days) of observation. Only one patient acquired an infection, and in this case, the infection developed towards the end of the observation period of 23 days. The OFPS (Model 110-4B, Camino Laboratories) was housed in a catheter and was inserted via a hole drilled in the skull and closed by a locking screw (schematic in Figure 7a). This study demonstrated easy and safe monitoring of ICP. In 2007, Bekar et al. [153] published an analysis of the risk factors of OFPS in intra-cranial pressure monitoring on 631 patients. They concluded that the ICP monitoring system could be safely used and that the infection risk is low (1.8%).

Chavko *et al.* [154] (2007) demonstrated an intra-cranial pressure measurement in generated blast waves using OFPS. The sensors were placed in the third cerebral ventricle of anaesthetized male rats. The pressures recorded were as high as 40 kPa and measured for several milliseconds. The rats were placed in pneumatic-driven shock tubes (schematic in Figure 7b), generating the blast waves. A 1 mm hole was drilled into the head of the rats, and the sensor, placed in a 23-gauge catheter, was inserted. Leonardi *et al.* [155,156] tested with the same sensor (FOP-MIV) and a similar experimental configuration the ICP of 25 rats.

An analysis of the transient response in a human skull, with exposure to blast waves, was published by Bir [157] in 2011. The shock waves produced a pressure up to 137.9 kPa. The OFPS (FOP-MIV, FISO) was placed in frozen human heads and placed in the tube. During 15 blast simulations, the sensors measured the pressure in four different areas of the brain. The experiments demonstrated the importance of sensor location, the intensity of the blast wave and the orientation of the head to the wave when undertaking sensor measurements in the brain.

**Urodynamics:** The pressure measurements undertaken during the course of urodynamic studies and which are relevant to the lower urinary tract (LUT) requires measurement in the urethra, bladder and abdomen. This analysis is important in order to diagnose bladder-related conditions [28,158]. Urodynamic analysis plays a key role as a method to localise pathological obstruction [159].

In 1993, Belville *et al.* [160] demonstrated the feasibility of a urodynamic system with OFPS (FST 200). The optical sensor was placed in a 1.6-mm (5 Fr) catheter, which was FDA approved for multiple usage. The sensor was based [160] on a diaphragm displacement technique with an intensity-modulated signal. The properties of the system demand a calibration cycle of 15 s before each use. The resolution in pressures measured was better than 100 Pa (1 cm H_2_O) up to a frequency of 50 Hz.

**Figure 8:**
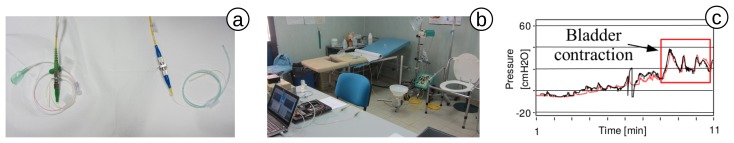
(**a**) Left: abdominal balloon catheter; right: bladder catheter; (**b**) examination chair with equipment; (**c**) urodynamic measurement.

Poeggel *et al.* [87] (2014) achieved *in vivo* bladder and abdominal measurements in patients, using a differential measurement technique, which allowed the simultaneous measurement of urodynamic pressure in a 1.6-mm (5 Fr) catheter, as well as abdominal pressure (Figure 8a,b). In a study published in 2015 [161] (Figure 8c), the technique was extended using an EFPI sensor with integrated FBG (*i.e.*, measuring pressure and temperature with a single sensor), creating an optical fibre pressure and temperature sensor (OFPTS). Furthermore, two sensors were placed in a single 1.6-mm (5 Fr) catheter, with a separation of 1 cm. This technique facilitated a true differential pressure measurement.

**Additional** *in vivo* pressure measurements were undertaken in humans and animals. Some of them are listed here, in order to demonstrate the range of use of optical fibre pressure sensors. These include measurements of intra-cochlear pressure (ear pressure) [162], dental pressure [163,164], intra-ocular pressure (IOP) [165,166] or vitreoretinal microsurgery [167,168].

A sensor for the dynamic assessment of the female pelvic floor function by intra-vaginal pressure was developed by Ferreira *et al.* [169]. The sensor (based on an FBG) was inserted into a silicon probe that measured radial muscle pressure and used this to interpret the axial load. The intra-abdominal pressure (IAP) of the glenohumeral joint was analysed by Inokuchi *et al.* [170]. The intra-muscular pressure (IMP) in rats was measured by Cottler *et al.* [171] and in the legs of four female and three male humans by Nilsson et al. [172]. Le *et al.* [173] developed a pressure sensor for tissue. In 2015, Roritz *et al.* [174] measured the pressure in the intervertebral disc pressure (IDP) of an anaesthetized sheep. The intra-discal pressure pattern was measured in the fifth lumbar disc, and this showed good agreement with previous results during spontaneous breathing. Furthermore, the lung pressure was measured in a different set of experiments. An OFPS (Camino 420 XP) was used in the endotracheal tube by De Blast *et al.* [175]. Intra-tracheal pressure was measured by Sondergaard *et al.* [176] and intra-oesophageal pressure by Hogan and Mintchev [177].

### 4.3. Research in Medical Optical Pressure Sensors: Ex Vivo

*Ex vivo* pressure measurements are measured outside of the body, disembowelled organs or in phantoms. The restrictions applied to these measurements are not as rigorous as for *in vivo* in humans or animals. Some recent experiments in this area are described below.

**Thermal ablation** is an invasive hyperthermia procedure: using a radio-frequency, microwave, laser or ultrasound source, using a micro-miniature applicator, delivered to the precise point of treatment. Using this technology, it is possible to generate a heat field *in vivo* with excellent localization capability. The radio frequency ablation (RFA) therefore has been successfully applied to many recent cancer treatments [178,179]. Using a percutaneous miniature applicator, RFA induces a heat field in excess of 3 °C/mm at the point of treatment. Cancer cells are euthanized as a function of temperature and the duration of its exposure [180].

Due to the lack of suitably-miniature and electrically-immune measurement equipment, it is not possible to implement pressure measurement in clinical RFA. The first pressure experiment in RFA is credited to Kotoh *et al.* [181] (2005), using an MEMS sensor positioned 3 cm from the ablation point. Previously, it was not possible to incorporate pressure sensing in the delivery of radio-frequency ablation due to the lack of a suitable sensing system.

**Figure 9:**
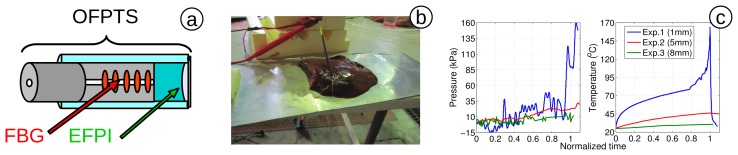
(**a**) OFPTS with EFPI and integrated FBG sensing element [183]; (**b**) liver phantom with OFPTS; and (**c**) pressure and temperature distribution in time. (adapted from Figures 21a and 24: Tosi, D., licensed under CC Attribution-Adapt Alike 3.0, 2015 [182])

Tosi *et al.* [86] (2014) performed the first pressure measurement *ex vivo* for RFA, with an OFPTS (Figure 9a) implementing a sensor on an animal liver phantom (Figure 9b). The sensing system used has the key advantage of having low thermal sensitivity, which allows operation at the point of ablation without the negative effects of large temporal and spatial temperature variations. A methodology for pressure measurement in RFA is reported in [182]. Pressure was recorded as 162 kPa (at 164 °C temperature), while in [86], a peak value of 750 kPa was recorded due to the encapsulation of the phantom (Figure 9c).

**Additional** *ex vivo* measurements of heart rate were recorded using optical fibre macro bending sensors on foot artery [184] or mounted around the arm [185]. FPI sensors for needle tip force sensing were tested on a medical skin phantom [186]. An *ex vivo* pressure test was also performed in lumbar IDP [187]. Smart devices have also been developed, where FBGs were inserted in clothes [188], beds [189,190] or furniture [191]. A wide variety of *ex vivo* or *in vitro* pressure measurements in other physiological locations have been successfully performed, including oesophageal pressure [192] and lung pressure during respiration [193].

### 4.4. Optical Pressure Sensors: Companies and Products

Companies that offer optical sensors [88,101,102,194–196] are listed in Table 4, demonstrating their potential for medical application [61,127,136,142,149,155,166,197–207].

## 5. Conclusions

The purpose of this review was to provide an update of the current state-of-the-art in optical fibre pressure sensors (OFPS) for use in the medical field. The sensors highlighted in this review are based on the principles of a Fabry–Perot interferometer (FPI) and fibre Bragg grating (FBG) techniques, whose characteristics and performance (e.g., range, sensitivity) are comparable, if not superior to commercially available electronic pressure sensors. However, instead of an electrical signal, the sensors modulate light. The absence of electrical signals makes the optical fibre sensors immune to radio frequency (RF) signals, which offer unique benefits, e.g., in harsh environments, such as MRI. The small diameter makes the sensor suitable for medical applications in volume-restricted areas, such as blood vessels or internal organs. The low attenuation of the optical single mode fibre (SMF) allows a potentially very long distance from the sensing element to the acquisition system, which is an advantage, e.g., in dangerous conditions, such as an epidemic patient in a contamination room without adequate equipment. Additionally, if a pressure sensor system were brought into such an area, it could be hard to remove, exchange or repair the system without taking additional risk. Furthermore, OFPS are inexpensive to produce, which makes them easily disposable. This may reduce the risk of infection, and since they are fabricated from glass, a disposable sensor has less impact on the environment when disposal is required. These above properties of OFS represent significant advantages, compared to electrical sensors, which makes them particularly well suited for medical application.

Furthermore, the human organism is a complex combination of a variety of organs, bones, joints and muscles, all of which have different pressure properties and measurement requirements. These requirements are often defined in standards, which are approved by authorizing institutions, such as FDA and ISO. Therefore, any optical sensor system has to be adapted to the needs of the specific medical examination. The sensitivity of FPI sensors can be easily adjusted by changing the thickness, material and diameter of the diaphragm. The change of sensitivity, as the main factor for determining pressure resolution, allows the adoption of the same sensor design for use in high-range or high-resolution sensors. With the inclusion of FBGs, it is possible to achieve single (e.g., for pressure and temperature) or multi-point measurements in a single 125–200-μm sensing element. OFPS have demonstrated stabilities up to 3 mmHg/23 days, which also allow accurate long-term measurements, which due to their size, can be fully *in vivo*. Their small size also allows the sensors to be placed in a standard catheter and, hence, for it to be guided to the point of interest, which avoids the use of over-/under-damping of water/air-filled catheters and gives a more accurate pressure signal.

Nevertheless, a high variety of *in vivo* and *ex vivo* applications have already been demonstrated and reviewed in this article. Optical pressure sensors for ICP and IABP have become the norm for clinical use in recent years. Furthermore, new technologies, such as the multipoint manometry catheter, based on an FBG array with up to 72 sensing elements, have the potential to create new gold standards. The small size allows housing of dual (or multiple) sensors in one catheter, which allows differential analysis as demonstrated in urodynamic measurements. Additionally, newer technologies, such as a radio-frequency RF ablation technology, have shown how optical fibre-based pressure sensors can satisfy modern medical demands, which other sensor technologies are not able to do. A summary of the main research impact, based on the number of publications, is included in Table 5. It demonstrates the current state of sensor technologies, how far the medical field of OFPS has penetrated into this application area, as well as the research impact. In particular, the latest test results captured in real medical environments demonstrate the excellent potential for future clinical use and emerging application areas for OFPS.

## Figures and Tables

**Table 1:** Classification and management of blood pressure [22].

**Blood Pressure (BP) Classification**	**Diastolic**	**Systolic**	**Treatment**
Normal:	<80 mmHg	<120 mmHg	Normal
Prehypertension:	80–89 mmHg	120–139 mmHg	No antihypertensive drug
Hypertension Stage 1	90–99 mmHg	140–159 mmHg	ACE, ARB, β-blocker
Hypertension Stage 2	>100 mmHg	>160 mmHg	2-Drug combination

**Table 2:** Collection of exemplary standards for medical pressure analysis. ICP, intra-cranial pressure; AAMI, Association for the Advancement of Medical Instrumentation; ERS, European Respiratory Society; ATS, American Thoracic Society.

**Area (Technique)**	**Body Part**	**Min. Pressure**	**Max. Pressure**	**Pressure Resolution**	**Sampling (Frequency)**	**Additional**	**Reference**
Cardiology (BP Monitoring)	Heart, Veins, Arteries	–4 kPa (–30 mmHg)	40 kPa (300 mmHg)	13 Pa (0.1 mmHg)	200 Hz	Volume restricted	AAMI BP22 [21]
Urology (Cystometry)	Bladder, Abdomen	0 Pa (0 cmH_2_O)	25 kPa (250 cmH_2_O)	50 Pa (0.5 cmH_2_O)	10 Hz	Differential measurement	Schaefer ^*1^ [28] and [29–31]
Neurology (ICP Monitoring)	Brain, Skull, Dural Tissue	0 Pa (0 mmHg)	13.3 kPa (100 mmHg)	260 Pa (2 mmHg)	-	Sterilization	AAMI NS28 [32], Andrew *et al.* [33]
Pulmonology (Transpulmonary)	Respiratory Tract, Lungs	–10 kPa (–100 cmH_2_O)	15 kPa (150 cmH_2_O)	2–40 Pa (4 mmH_2_O)	200 Hz (10 Hz)	Temperature, humidity	ERS/ATS [34,35] Bensenor [36]
Gastroenterology (Manometry)	Stomach, Colon	0 Pa (0 mmHg)	13.3 kPa (100 mmHg)	-	8 Hz	Multi probes (6 or more)	RAO *et al.* [37] Cross-Adame [38]
Ophthalmology (Tonometry)	Eyes	0 Pa (0 cmH_2_O)	8 kPa (60 mmHg)	13 Pa (0.1 mmHg)	100 Hz	Volume restricted	Weinreb [39] ISO 8612 [40]
Rheumatology	Muscle, Bones, Spine	0 kN ^*2^	3 kN ^*2^	-	-	High pressure	ISO 14242-2 [41]
Cancer Treatment (Ablation)	Full Body	0 kPa ^*3^	200 kPa ^*3^	-	-	Temperature, RF-field	

*1 The recommendation by Schaefer *et al.* was also used by the NHS Purchasing and Supply Agency (U.K.), for their official buyers guide for urodynamic systems (CEP08045 ) [42].

*2 Instead of pressure, the weight distribution is of interest. It is mentioned here, as well, since the same principles can be used.

*3 The lack of research in this field has complicated a determined standardization. The values are based on observation by preliminary tests.

**Table 3:** Electrical pressure sensors available on the market.

**Company**	**Merit Sensor**	**Elcam Medical**	**MEMSCAP**	**Me. Specialities**
Sensor Name	BP series [50]	Sense-IT [51]	SP854 [52]	1620 [53]

Min. Pressure	–4 kPa (–30 mmHg)	–4 kPa (–30 mmHg)	–80 kPa (–600 mmHg)	6.66 kPa (50 mmHg)
Max. Pressure	40 kPa (300 mmHg)	40 kPa (300 mmHg)	93 kPa (700 mmHg)	40 kPa (300 mmHg)
Over Pressure	862 kPa (125 Psi)	862 kPa (6465 mmHg)	1.3 MPa (10 MHg)	862 kPa (125 Psi)

Frequency	1.2 kHz	1.2 kHz	300 Hz	1.2k Hz

Drift	0.125 mmHg·h^−1^	0.125 mmHg·h^−1^	-	0.25 mmHg·h^−1^

Compliant	AAMI, RoHS	510(k), AAMI, CE, ISO 2009, 10993-1	OEM-Part	AAMI, RoHS

Restriction of Hazardous Substances (RoHS); Original equipment manufacturer (OEM); Measurement (Me.) Specialities.

**Table 4:** Optical pressure sensors available on the market.

**Company**	**Samba**	**FISO**	**Camino**	**Opsens**	**RJC Enterprise**	**Maquet**
Sensor Name	Preclin 420/360 Transducer [194]	FOP-MIV (R1) [101]	Model 110-4B [195]	OPP-M25 [88]	Model 40 [102]	CS300 [196]
Min. Pressure	–5 kPa (–37.5 mmHg)	–40 kPa (–300 mmHg)	–1.3 kPa (–10 mmHg)	–6.66 kPa (–50 mmHg)	66 kPa (500 mmHg)	0 kPa (0 mmHg)
Max. Pressure	35 kPa (262 mmHg)	40 kPa (300 mmHg)	16.7 kPa (125 mmHg)	40 kPa (300 mmHg)	133 kPa (1000 mmHg)	40 kPa (300 mmHg)
Pr. Resolution	10 Pa (0.07 mmHg)	40 Pa (0.3 mmHg)	-	66 Pa (0.5 mmHg)	< 0.1 mmHg	-
Over Pressure	-	530 kPa (4000 mmHg)	166.7 kPa (1250 mmHg)	533 kPa (4000 mmHg)	-	-
Frequency	40 kHz	250 Hz	120 Hz	250 Hz	1 kHz	26 Hz
Diameter (in Catheter)	0.36–0.42 mm	0.55 mm	1.35 mm (4 Fr)	0.25 mm	0.17 mm	2.33 mm (7 Fr)
Approved	-	FDA	FDA	-	(AAMI BP)	FDA
Tested for	left ventricle [197] ICP [198,199] IDP [200]	IAP ^*1^ [201], pharyngeal ^*1^ [127], ICP [155,202]	ICP ^*1^ [203,204] IMP ^*1^ [61,205] IAP [206]	IAP [149] IOP [166]	AP [136]	IABP ^*1^ [142,207]

In some cases, a different sensor by the same company was used. However, the references demonstrate the feasibility; Measurements in a human are marked with (^*1^). IDP, intervertebral disc pressure; IAP, intra-articular pressure; IOP, intra-ocular pressure; IABP, intra-aortic balloon pumping; Pr., Pressure.

**Table 5:** Main publications, sorted by the number of publications in this review.

**Medical Area**	**Modulation Type**	**Place**	**Temperature Compensation**	**Sensor State**	**Already Explored**	**Research Impact**	**Publications**
Cardiology	Intensity, FPI, FBG	*in vivo*, *ex vivo*	No	100 % ^*1^	70 %	70 %	18
Neurology	Intensity, FPI	*in vivo*	No	100 % ^*1^	70 %	60 %	9
Gastroenterology	FPI, FBG	*in vivo*	Possible	70 %	50 %	60 %	7
Pulmonology	Intensity	*in vivo, ex vivo*	No	30 %	20 %	20 %	4
Ophthalmology	FPI, FBG	*ex vivo*	No	40 %	20 %	30 %	4
Urology	Intensity, FPI + FBG	*in vivo*	Yes	60 %	30 %	40 %	3
Rheumatology	Intensity, FBG	*in vivo, ex vivo*	No	60 %	30 %	40 %	3
RF ablation	FPI + FBG	*ex vivo*	Yes	30 %	20 %	60 %	2

*1 Medical sensors existing on the market and used by clinicians for medical examinations.
